# Modulation of Innate Immune Responses by Influenza-Specific Ovine Polyclonal Antibodies Used for Prophylaxis

**DOI:** 10.1371/journal.pone.0089674

**Published:** 2014-02-28

**Authors:** Catherine Rinaldi, William J. Penhale, Philip A. Stumbles, Guan Tay, Cassandra M. Berry

**Affiliations:** 1 Centre for Forensic Science, The University of Western Australia, Nedlands, Western Australia, Australia; 2 Molecular and Biomedical Sciences, School of Veterinary and Life Sciences, Murdoch University, Murdoch, Western Australia, Australia; University of Georgia, United States of America

## Abstract

In the event of a novel influenza A virus pandemic, prophylaxis mediated by antibodies provides an adjunct control option to vaccines and antivirals. This strategy is particularly pertinent to unvaccinated populations at risk during the lag time to produce and distribute an effective vaccine. Therefore, development of effective prophylactic therapies is of high importance. Although previous approaches have used systemic delivery of monoclonal antibodies or convalescent sera, available supply is a serious limitation. Here, we have investigated intranasal delivery of influenza-specific ovine polyclonal IgG antibodies for their efficacy against homologous influenza virus challenge in a mouse model. Both influenza-specific IgG and F(ab’)_2_ reduced clinical scores, body weight loss and lung viral loads in mice treated 1 hour before virus exposure. Full protection from disease was also observed when antibody was delivered up to 3 days prior to virus infection. Furthermore, effective prophylaxis was independent of a strong innate immune response. This strategy presents a further option for prophylactic intervention against influenza A virus using ruminants to generate a bulk supply that could potentially be used in a pandemic setting, to slow virus transmission and reduce morbidity associated with a high cytokine phenotype.

## Introduction

A serious pandemic threat lies with a new emerging influenza A virus that is highly virulent and transmissible and to which humans have weak or no prior immunity [1], [2], [3], [4]. Additionally, seasonal influenza afflicts millions of people each year and is responsible for over 250,000 annual deaths, despite available vaccination and antiviral drugs [5]. Mortality rates for zoonotic avian influenza viruses have reached 60% worldwide for H5N1 and approximately 30% for a newly emerged pathogenic H7N9 strain in China [6], [7]. The lag time required to produce and distribute vaccines against influenza once a newly emergent virus strain has been identified is likely to render many unprotected and at risk. Indeed, during the last pandemic, the novel 2009 H1N1pdm swine influenza virus was able to rapidly disseminate worldwide in six weeks despite a record rate of manufacture of a strain-specific vaccine, albeit with doses available to cover only 10% of the world's population. A new approach is needed, particularly one supplementing those aspects where influenza control by vaccination is constrained, including the variable immunogenicity and protracted time to develop full immunity post-vaccination in individuals. Wide scale deployment of a prophylactic able to provide immediate protection regardless of age and immune memory, even if only temporarily, could dramatically reduce the number of cases by preventing/reducing virus infection and thereby have a significant impact on virus spread.

To augment the pandemic vaccination approach [8], adjunct regimes of passive immunity mediated by antibodies specific for influenza virus have been investigated [9], [10], [11]. Convalescent human sera from survivors of the H1N1 Spanish Flu pandemic have been effective in reducing the mortality rate but supply is not readily available [12], [13]. Although passive immunotherapies using human monoclonal antibodies specific for viral HA and NA subtypes and conserved M2 have demonstrated efficacy in animals, equivalent to the antiviral drugs amantadine, oseltamivir and zanamivir [14], [15], [16], their production and distribution requirements would likely be impractical for emergency use and potentially drive virus escape mutations, thereby limiting their efficacy [17]. Polyclonal antibodies generated in ruminants by vaccination with influenza A viruses have been shown to be prophylactic in animal models of influenza with reduced lung virus titres and increased survival upon virus challenge [18], [19], [20]. Most often the delivery of antibody has been systemic via the intraperitoneal or intravenous routes requiring large doses. However, benefits of intranasal administration of antibodies are dose sparing with targeting to the primary site of natural exposure to influenza viruses. Topical delivery of antibodies to the respiratory tract mucosa may also offer advantages of simple self-administration by nasal spray. Moreover, production of neutralizing antibodies against different influenza A virus groups could be stockpiled for immediate use in controlling virus outbreaks [21].

Although intranasal delivery of influenza virus-specific antibodies derived from bovine colostrum has been shown to provide protection against influenza A virus infection, their influence on host immune responses have not been reported [22]. Homeostasis is paramount in the lung [23], however, during virus infection, the rapid induction of innate cytokine responses, including the type I interferons, are essential for effective influenza-specific immunity [24], [25]. Interferon-α/β have potent and direct antiviral properties in addition to modulatory effects on immune responses but hyperinduction of cytokines in the respiratory tract leads to acute lung injury and acute respiratory distress syndrome in some individuals, which can be fatal [26], [27]. This was most evident in the 1918 Spanish Flu pandemic, especially in healthy young adults with a robust immune system [28]. A significant benefit would be to provide antiviral immunity whilst protecting predisposed individuals and those in age groups susceptible to severe illness caused by such “cytokine storms” induced by their inflammatory responses in exposure to lethal influenza A virus strains. In this study, we have investigated the efficacy and influence on the innate immune response of influenza-specific polyclonal antibodies, raised in sheep, against influenza in a mouse model. We report that a single topical administration of virus-specific polyclonal IgG elicits complete protection when delivered up to 3 days before virus exposure, largely independent of the induction of innate cytokine pathways.

## Materials and Methods

### Animal Ethics

Animal experimentation was compliant with the Australian Code of Practice for the Care and Use of Animals for Scientific Purposes. All mouse (*Mus musculus*) experimentation was conducted with approval from Murdoch University (R2229/09) and The University of Western Australia (RA/3/100/89) Animal Ethics Committees. All sheep (*Ovis aries*) experimentation was performed with ethics approval from Murdoch University (R2178/08) and conducted at the Murdoch University Veterinary Farm.

### Influenza A Virus Antigen Preparations

Antigen preparations for immunization of sheep were prepared from influenza A/Puerto Rico/8/34 (PR8) virus obtained from ATCC and propagated in confluent Madin Darby Canine Kidney (MDCK) cells grown in Dulbecco's modified Eagle's medium (DMEM, Sigma, MO, USA) with 0.5% fetal calf serum (Gibco, Aukland, NZ) and 5µg/mL trypsin, penicillin (100 U/mL) and streptomycin (100µg/mL) (Sigma, MO, USA). Crude tissue culture supernatant containing virus was inactivated with formalin (Sigma, MO, USA) for 65 hrs at 37°C and clarification by centrifugation at 5,250× g for 5 min. Bulk tissue culture supernatant containing virus was also concentrated using a 10 kDa membrane cut-off Amicon (Millipore, Cork, Ireland), pelleted by centrifugation at 114,000× g for 90 min at 4°C and purified by sucrose gradient ultracentrifugation [29]. The purified virus was then resuspended in 1 mL 0.05 M sodium acetate/2 mM sodium chloride/0.2 mM EDTA (pH 7.0) and detergent-disrupted by treatment with 0.01% Triton X-100 (Sigma, MO, USA) overnight at 4°C and then diluted in PBS. Virus antigens were prepared as water-in-oil emulsions with Titermax Gold adjuvant (CytRx Corporation, GA, USA) and contained 0.3 mg protein/mL per vaccine dose as determined by the Bradford protein assay.

### Ovine Serum and Whey Samples

Lactating ewes were immunized with either inactivated virus antigen or purified detergent-disrupted virus antigen preparations in the gluteal muscle mass of the hind leg, followed by a further two boosters at day 14 and days 21–28. Blood and milk was collected at days 45–58 from immunized sheep and non-immunized sheep as a control. Both serum and whey were stored at −20°C.

### Antibody Purification

IgG purification from serum was performed using Protein-G affinity chromatography, with PBS (pH 7.3) as running buffer and 0.1 M Glycine (pH 2–3) as elution buffer. Eluted protein fractions were neutralized with 1 M Tris (pH 7.5–9) and then diafiltrated with PBS using 10 kDa cut-off membranes. Protein was concentrated using 50 kDa cut-off Amicon ultrafiltration membrane and endotoxins removed with Detoxi-Gel™ columns (Thermo Scientific, Rockford, USA). For F(ab’)_2_ preparation, purified IgG was dialyzed against 0.2 M sodium acetate (pH4.5) for 4 hr at 4°C and digested (1 mg/ml) with pepsin (0.1 mg/ml) for 24 h at 37°C, after which the pH was increased to 8.0 with 2 M Tris base and the resulting F(ab’)_2_ fragments dialysed against PBS overnight at 4°C. Purified IgG and pepsin digested IgG were characterized by SDS-PAGE. Under reducing conditions, two major bands were seen for IgG, representing H (50 kDa) and L (25 kDa) chains, whereas the F(ab’)_2_ preparation showed L (25 kDa) chains only (data not shown). Protein concentrations were determined by absorbance at 595 nm wavelength by the Bradford method using colorimetric protein assay (Bio-Rad, CA, USA) and spectrophotometry.

### Hemagglutination Inhibition (HI) Assay

HI assays were performed according to standard protocols [30] using receptor destroying enzyme, 4 hemaggluninating units (HAU) of influenza PR8 virus and 1% chicken erythrocytes in round-bottom microtitre plates (Falcon, USA). Titres were expressed as the reciprocal of the highest dilution of antibody sample that inhibited hemagglutination.

### Neuraminidase Inhibition (NI) Assay

The NI assay was performed as previously described with a 1 hr incubation of 25µL anti-serum and 25µL influenza PR8 virus at room temperature prior to neuraminidase assay using fetuin and periodate, arsenite and thiobarbituric acid reagents in 96-well tissue culture plates [31], [32].

### Microneutralization (MN) Assay

Determination of virus neutralization titres of heat-inactivated whey and serum samples was performed using standard protocols and 10^2^TCID_50_ influenza PR8 virus [33]. MDCK monolayers were grown to confluency in DMEM and 10% FCS before washing twice with PBS. Serially diluted antibody preparations were mixed with an equal volume of virus and incubated for 60 mins at 37°C and 5%CO_2_. MDCK cells were then incubated in duplicate, with 100µL of virus/antibody mixtures diluted in DMEM with 0.5% FCS and 5µg/mL trypsin, for 72 hours at 37°C and 5%CO_2_. Cytopathic effect was scored and the MN titre expressed as the reciprocal of the highest dilution with 100% inhibition of cytopathic effect.

### Hemagglutination (HA) Assay

The hemagglutination assay (HA) was performed using standard protocols described by the World Health Organisation [30]. Briefly, PR8 virus was serially diluted in PBS/0.1% FCS and incubated with 1% chicken red blood cells in U-bottom shaped microtitre plates at 4°C for 60 minutes. 1 HAU was defined as the reciprocal of the last dilution to provide complete hemagglutination of the RBC.

### Antibody ELISA

Mouse anti-sheep antibodies were determined by ELISA [34] using sheep IgG (1µg/well, Abcam, Cambridge, MA, USA) to coat plates overnight at 4°C. Plates were blocked with 1% ovalbumin before addition of mouse serum samples (day 14 p.i.) that were serially diluted in PBS-Tween 20. Antibody reactivity was detected using conjugated anti-mouse IgG alkaline phosphatase and 4-nitrophenyl phosphate disodium salt hexahydrate diluted with diethanolamine buffer as substrate. Absorbance was read at 405 nm by spectrophotometry.

### Treatment Protocol and Challenge Infection of Mice

Specific pathogen-free inbred BALB/c mice (6–10 weeks old) were obtained from the Animal Resource Centre (Murdoch, WA, Australia) and housed at the Animal House facility at Murdoch University. Mice were lightly anaesthetized with methoxyfluorane before infection with 25µL influenza PR8 virus (10^2^TCID_50_) by the intranasal route via administration by micropipette to the nares. For antibody prophylaxis, mice were given 25µL antibody or PBS by the intranasal route 1 hr, 1 day, 3 days or 7 days before virus infection or 1 day after virus infection. Mice were killed by lethal dose of sodium pentobarbitol 3 days after virus infection for determination of virus titres in the lungs or monitored daily for clinical symptoms (ranging from 0–5) and weight changes over 2 weeks p.i. Clinical scores were defined as 0) healthy, 1) barely ruffled fur, 2) fur ruffled but active, 3) fur ruffled and inactive, 4) fur ruffled, inactive and hunched appearance, and 5) dead. Mice were culled if they had lost >25% body weight and reached a clinical score of 4, due to ethical considerations. Mice were bled at day 14 p.i. and serum stored at −20°C.

### Lung Characterization

Virus loads in the lungs were determined by TCID_50_ assay using lung homogenates (20%w/v) and MDCK cells. The log_10_ TCID_50_ titre was calculated according to the Reed and Muench method [35]. Bronchioalveolar lavage (BAL) was performed using 0.5 mL PBS with three washings for each animal. BAL fluid samples were centrifuged at 450× g for 10 mins and the supernatant stored at −80°C.

### Cytokine Assays

Type I interferons in the BAL fluid samples were quantitated in the bioassay as previously described [36] using 70% confluent L929 cells grown in DMEM and 10% FCS at 37°C and 5%CO_2_. Cells were washed twice in PBS and then the IFN standard (Universal IFN, PBL Interferon Source, NJ, USA) or samples serially diluted in DMEM and 1% FCS were added in duplicate to the cells and incubated overnight at 37°C and 5%CO_2_. Following a further 24 hr incubation with encephalomyocarditis virus, a reduction in cytopathic effect (CPE) was indicative of antiviral activity and endpoint titres determined by the highest dilution resulting in 50% reduction of CPE.

Flow cytometry was used to determine IFN-γ, IL-1β, TNF-α, IL-6 and CXCL1 levels in the BAL fluid samples by cytokine bead array according to manufacturer's instructions (BD, MD, USA). Fluorescence was measured using the FACSCanto II and FACSDiva V6.1.2 (BD Biosciences) software. Data was analyzed using FCAP Array V3.0 software based on 5 parameter logistic curve fits.

### Statistical Analysis

Statistical analyses were performed using ANOVA with Tukey's multiple comparison test (Prism V5 GraphPad Software Inc., CA. USA). P values <0.05 were considered significant and are shown for comparisons of treatment groups with PBS controls unless stated otherwise.

## Results

### Immunogenicity of whole inactivated and split influenza PR8 virus vaccines in sheep

Advantages of tissue culture-based vaccines are that most influenza A virus strains grow well in cell culture without the need for extensive reassortment, which is required to produce seed viruses, and have a shorter scale-up period than egg-based systems [37], [38]. As different vaccine formulations can vary in efficacy according to species, we evaluated the immunogenicity of several PR8 H1N1 virus vaccine types prepared using cell culture systems on antibody production in sheep. Sheep were chosen as a suitable alternative ruminant to more expensive and larger cattle while allowing proof-of-concept studies in influenza prophylaxis using a mouse model. Thus in order to evaluate the immunogenicity of different virus antigen preparations, we examined the effect of whole inactivated virus and detergent-disrupted (split) virus on the induction of a virus-specific antibody response in sheep. A number of mechanisms including binding to HA and NA are required for neutralization of influenza A viruses [39]. For this reason we used HI, NI and MN assays to determine ovine antibody specificity to influenza PR8 virus. HI and MN antibody titres in serum and whey samples were generally higher in response to split virus antigen than the inactivated virus antigen (Table 1), likely dependent on HA protein concentrations. Only the split virus induced antibodies in the sera with positive NI activity to homologous PR8 virus, possibly due to higher concentrations of the NA antigen component in the purified preparation.

**Table 1:** Antibody titres to influenza A/PR/8/34 (H1N1) in sheep immunized with either whole inactivated or split PR8 virus.

Virus^a^	Sample	HI^b^	MN^c^	NI^d^
Inactivated virus	Serum	20, 320, 80	40, 640, 80	-, -, -
	Whey	<10, 10, 10	<10, <10, <10	ND
Split virus	Serum	640, 80	12800, 6400	+, +
	Whey	160, <10	12800, 100	ND

a Sheep were immunized with inactivated whole influenza A PR8/34 virus (n = 3) or purified split PR8 virus (n = 2) with adjuvant (Titermax Gold) at weeks 0, 2 and 3–4 and samples collected at weeks 6 and 8, respectively.

b H1-specific antibody titres in sera and whey determined by HI assay using homologous virus as antigen. Data expressed as reciprocal endpoint dilution of sample from individual sheep.

c Microneutralization titres in sera and whey determined by microneutralization assay using homologous virus. Data expressed as reciprocal endpoint dilution of sample from individual sheep.

d N1-specific antibody reactivity in sera determined by NI assay using homologous virus as antigen and expressed as (+) positive or (-) negative for individual sheep.

### Virus neutralization by PR8-specific ovine IgG *in vitro*

A number of antibody isotypes are found in the humoral response to influenza virus infection, but IgG plays a predominant role in virus neutralization [40], [41]. To evaluate the contribution of ovine IgG in neutralization of virus, IgG was purified using protein G affinity chromatography. Purified IgG from the split virus-immunized sheep was found to have a higher MN titre (6400) than the serum from the inactivated virus immunized sheep (320), however both purified IgG preparations had a reduced MN titre than whole serum preparations, likely due to some loss during the column chromatography process.

### Prophylactic efficacy of PR8-specific ovine IgG

**Figure 1:**
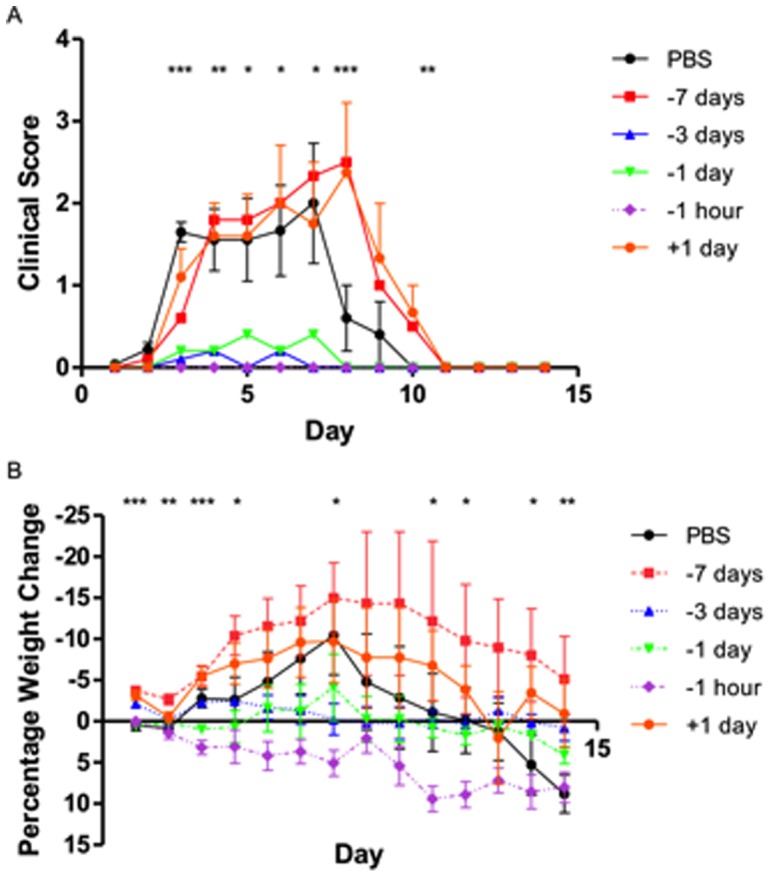
Protection of mice against influenza H1N1 virus with ovine IgG antibodies. Lightly anaesthetized female BALB/c mice (n = 10) were given 125µg IgG from split virus-immunized sheep or PBS by the intranasal route one hour, 1 day, 3 days, 7 days before or 1 day after i.n. challenge with 10^2^TCID_50_ PR8 and monitored daily for clinical illness and body weight loss. (**a**) Clinical score and (**b**) percentage weight change data are representative of four separate experiments with mean ± SEM shown. *P≤0.05, **P≤0.01, ***P≤0.001 represent comparisons between antibody-treated (-1 hr) and PBS control groups.

Antibody responses to influenza virus are important correlates of protection against influenza infection *in vivo*. Since, prophylactic use of heterologous antibody to provide passive immunity in a different species to that of the antibody source has been successful, we evaluated the effect of ovine antibody on influenza virus replication in the mouse model. We compared the effects of intranasal instillation of neutralizing virus-specific sheep IgG with PBS as a control, on protection of mice challenged by the same intranasal route with a sublethal dose of H1N1 influenza PR8 virus. The antibody dose of 125µg was chosen because it was known to prevent replication of 10^2^TCID_50_ influenza PR8 virus *in vitro* as determined by the MN assay. Clinical scores and body weight loss were examined daily during the first 2 weeks post-viral challenge. PBS treatment did not significantly reduce weight loss and clinical illness following virus challenge (Figure 1). In contrast, neutralizing virus-specific IgG preparations elicited reduced weight loss and lower clinical scores, even when delivered up to 3 days before virus challenge but was not effective when applied at 7 days before or 1 day after virus exposure.

**Figure 2:**
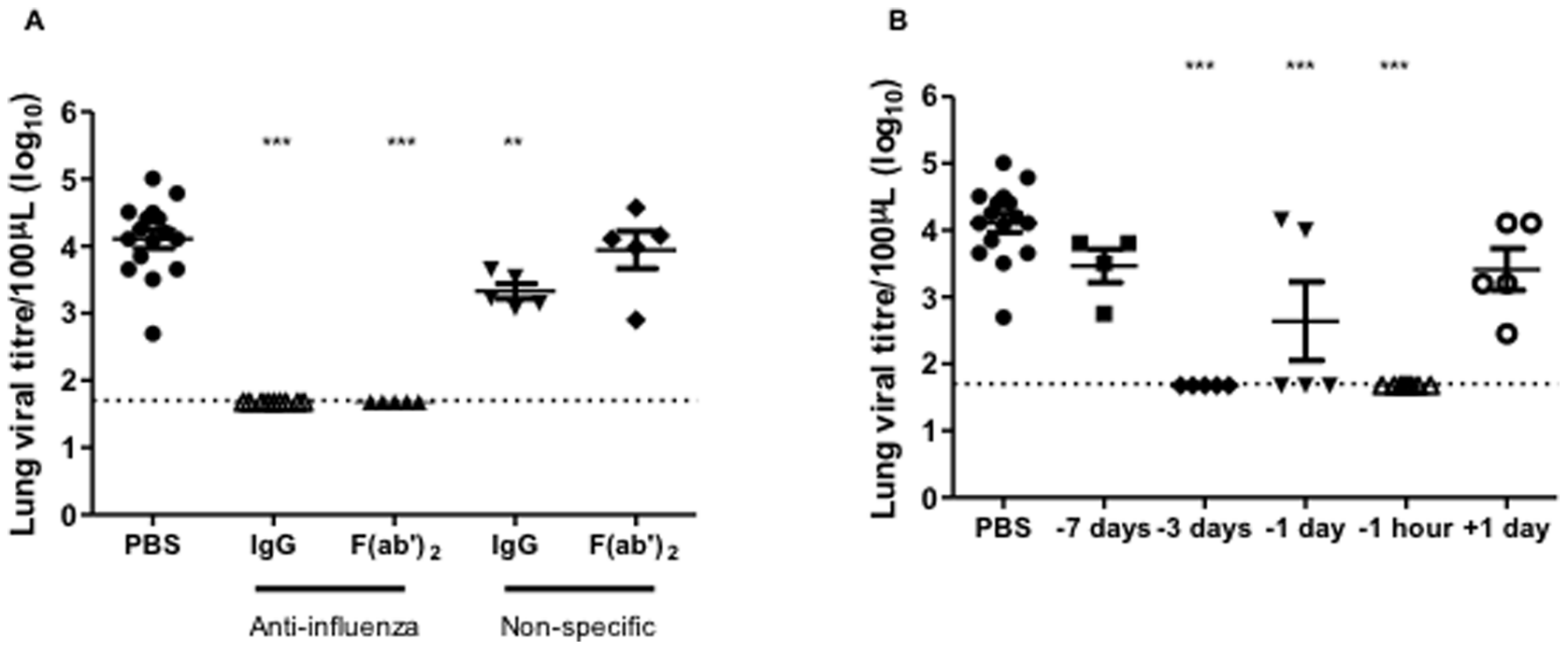
Lung viral titres in ovine IgG protected mice. Lightly anaesthetized female BALB/c mice (n = 5–16) were given (**a**) 125µg IgG or F(ab’)_2_ from split virus-immunized sheep, PBS or non-specific IgG or F(ab’)_2_ by the intranasal route one hour before i.n. challenge with 10^2^TCID_50_ PR8. (**b**) Mice were similarly given 125µg IgG from split virus-immunized sheep 1 day, 3 days, 7 days before or 1 day after virus challenge. Lung viral loads at day 3 p.i. were quantitated by TCID_50_ assay and individual titres and mean ± SEM are shown. Results are representative of four separate experiments and the dashed line shows the limit of detection. ** P≤0.01, ***P≤0.001 represent comparisons between antibody-treated and PBS control groups.

Since topical delivery of virus-specific antibody to the mucosal surface of the respiratory tract is a relevant site for protection from exposure to replicating virus, we next examined the ability of antibody treatment to reduce lung viral loads at day 3, a time of peak virus replication. Both the neutralizing IgG and F(ab’)_2_ preparations derived from the split virus immunized sheep significantly reduced lung viral loads in challenged mice when antibody was administered 1 hour before virus challenge in contrast to high viral loads in the lungs of PBS and non-specific F(ab’)_2_ treated animals (Figure 2a). Partial protection was observed in animals treated with non-specific IgG, similar to that previously reported for bovine IgG [22]. Furthermore, clearance of virus infection was evident in most of the mice given influenza specific-IgG at 3 days and 1 day before but not at 7 days before or 1 day after virus challenge (Figure 2b). Therefore, prophylaxis with topical administration of neutralizing IgG antibodies up to 3 days before virus exposure can effectively control influenza.

### Prevention of interferon responses in PR8-specific ovine IgG-protected mice

**Figure 3:**
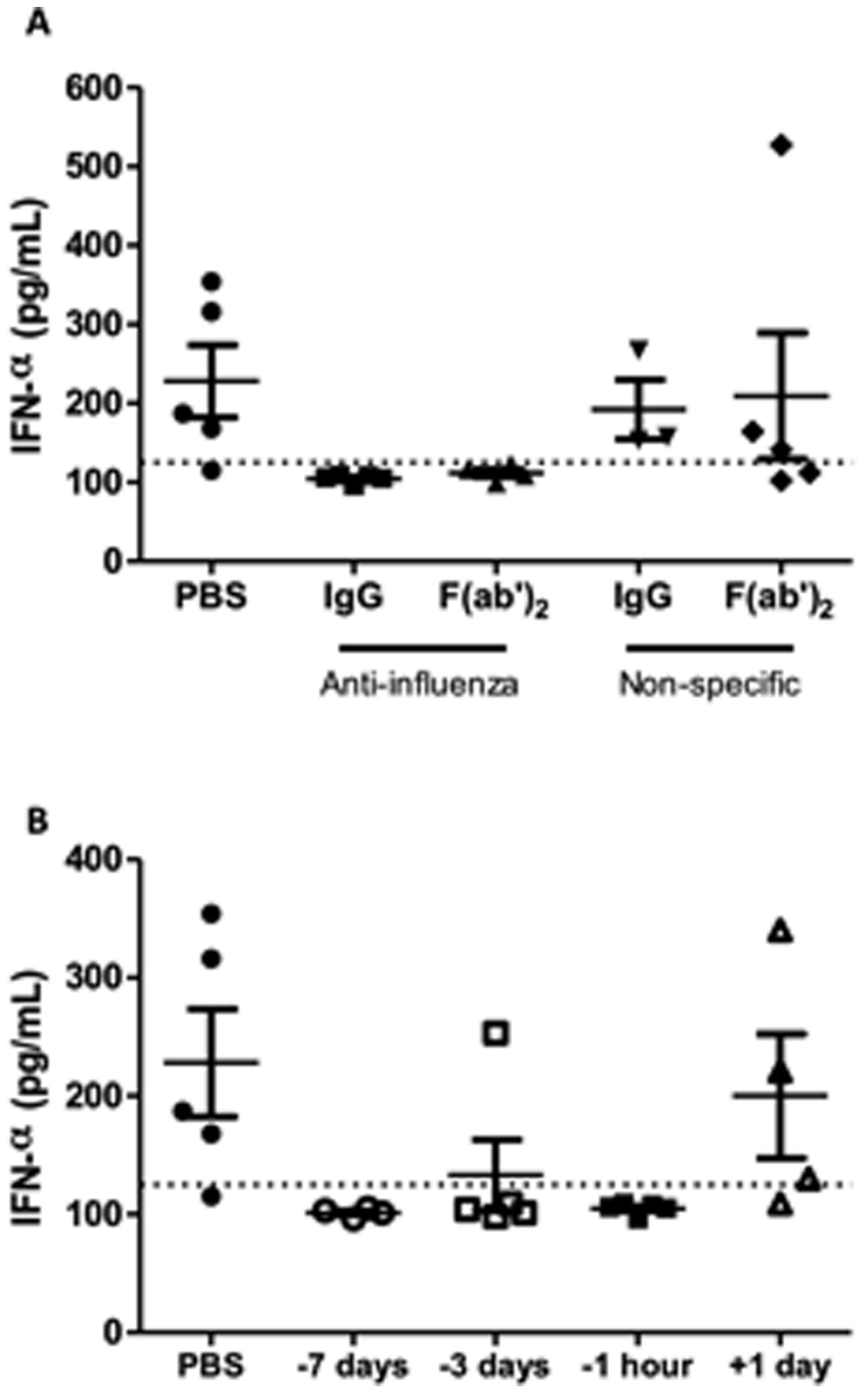
Interferon-α responses in the BAL samples of ovine IgG treated mice. Lightly anaesthetized female BALB/c mice (n = 5) were given (**a**) 125µg IgG or F(ab’)_2_ from split virus-immunized sheep, PBS or non-influenza specific control IgG or F(ab’)_2_ by the intranasal route one hour before i.n. challenge with 10^2^TCID_50_ PR8. (**b**) Mice were similarly given 125µg IgG from split virus-immunized sheep at 3 days before, 7 days before or 1 day after virus challenge. PBS was given one hour before virus infection. Results are representative of two separate experiments. Interferon-α responses in the BAL fluid samples collected at day 3 p.i. were quantitated by bioassay and individual titres and mean ± SEM are shown. The dashed line shows limit of detection.

Although high neutralizing IgG from sheep was shown to neutralize PR8 virus *in vitro* and prevent virus replication *in vivo*, the IgG-neutralized virus may be able to stimulate host innate immune responses that rapidly clear the virus. Recognition of foreign antigens through PAMP molecular encounters can lead to the early induction of innate interferon responses, which have potent antiviral properties [42], [43], [44]. We investigated contribution of the interferon-α response to the reduction of lung viral loads at day 3 p.i. in antibody-protected mice. Although a strong type I interferon response was found in the BAL samples taken at day 3 p.i. of most of the PBS-treated animals and mice given influenza non-specific IgG or F(ab’)_2_, complete abrogation of the type I IFN response was observed in animals given 125µg of the neutralizing IgG or (Fab’)_2_ derived from the split virus immunized sheep one hour before virus challenge (Figure 3a). The timing of antibody prophylaxis relative to virus challenge influenced the induction of a type I IFN response in the mice (Figure 3b). Reduced mean titres of IFN-α were found at day 3 p.i. of mice treated with high neutralizing antibody at 7 and 3 days before virus infection, despite some animals showing virus replication. However, some animals showed a type I IFN response in the BAL fluid samples when given virus-specific IgG one day after virus infection. Taken together, these results suggest that pre-exposure antibody treatment has potential value in neutralizing virus and clearing the infection during the acute stages, largely without a strong type I IFN response.

**Figure 4:**
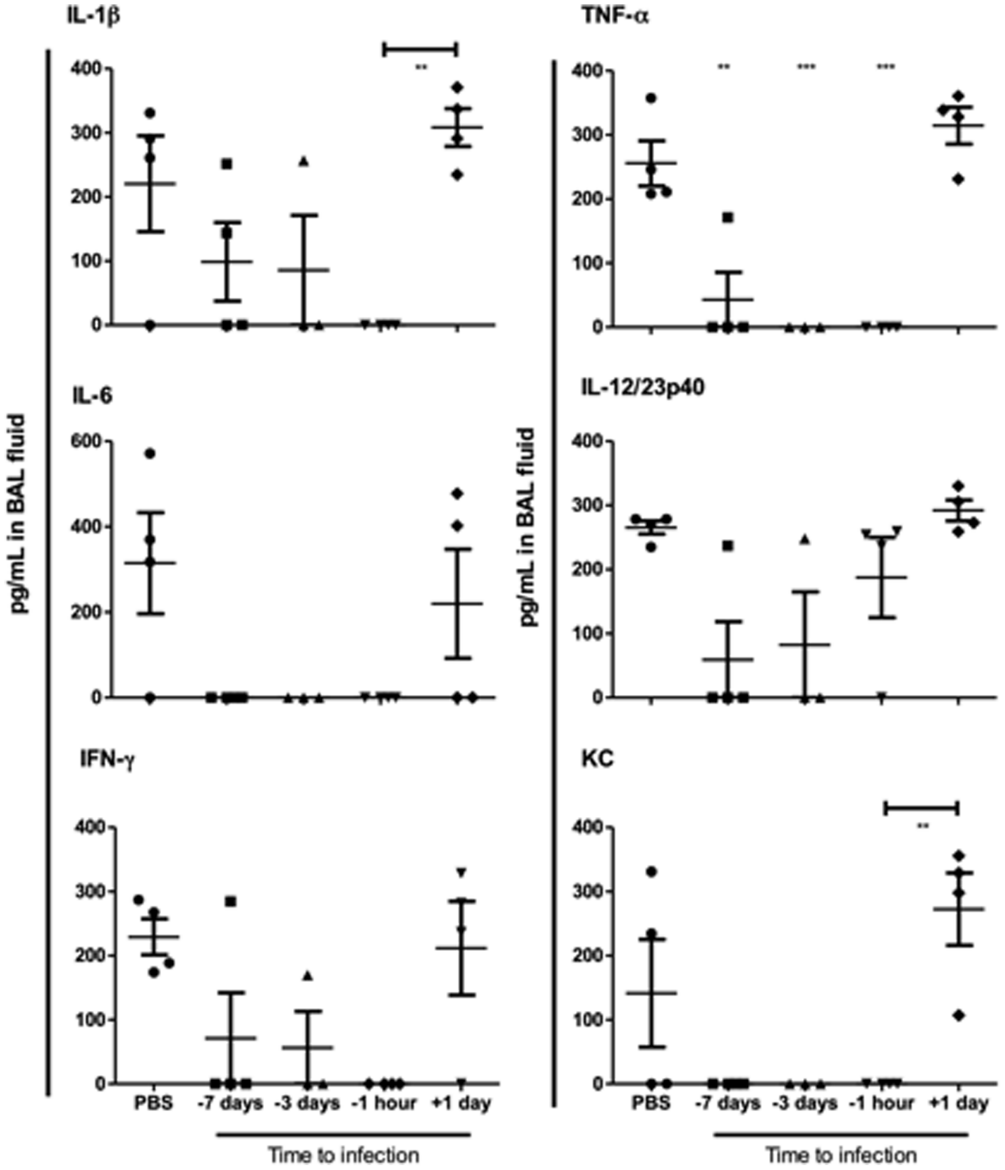
Cytokine responses in the BAL samples of ovine IgG protected mice. Lightly anaesthetized female BALB/c mice (n = 4) were given 125µg IgG from split virus-immunized sheep or PBS by the intranasal route one hour, 1 day, 3 days, 7 days before or 1 day after i.n. challenge with 10^2^TCID_50_ PR8. Cytokines measured by flow cytometry using cytokine bead arrays for IL1-β, TNF-α, IL-6, IL-12, IFN-γ and KC (CXCL1). Results are representative of four separate experiments with individual titres and the mean ± SEM shown. **P≤0.01, ***P≤0.001 represent comparisons between antibody-treated and PBS control groups. The bar with asterisks denotes significance levels between the specified (+1 day and -1 hr) antibody-treated groups.

Type II IFN levels during influenza virus infection largely indicate T cell activation and can be utilized as a biomarker for cell-mediated immune responses [45]. IFN-γ was measured by flow cytometry in the BAL fluid samples taken at day 3 p.i. Mice treated with neutralizing IgG derived from the split virus immunized sheep at one hour before virus exposure had no detectable IFN-γ levels (Figure 4). Only one animal in each group given virus-specific IgG treatment 3 and 7 days prior to virus challenge produced an IFN-γ response, whereas 3/4 animals in the group treated 1 day post-virus challenge, mounted a type II IFN response. In contrast to untreated animals, the cytokines IL-1β, TNF-α and IL-6 were not detected in BAL fluid samples at day 3 post-virus challenge of all animals pretreated with neutralizing IgG one hour before virus exposure (Fig. 4). In addition, TNF-α levels were significantly reduced when IgG was given either 7 or 3 days before virus challenge. The trend of lower IL-1β IL-6, IL-12 and CXCL1 (KC) levels was also observed in mice given antibody prophylactically, although not reaching significance. However, antibody delivered 1 day after virus exposure did not significantly change the cytokine levels for IL-1β, TNF-α, IL-6, IL-12 or CXCL1 in these unprotected mice.

### Host antibody responses to PR8 and sheep Ig

**Figure 5:**
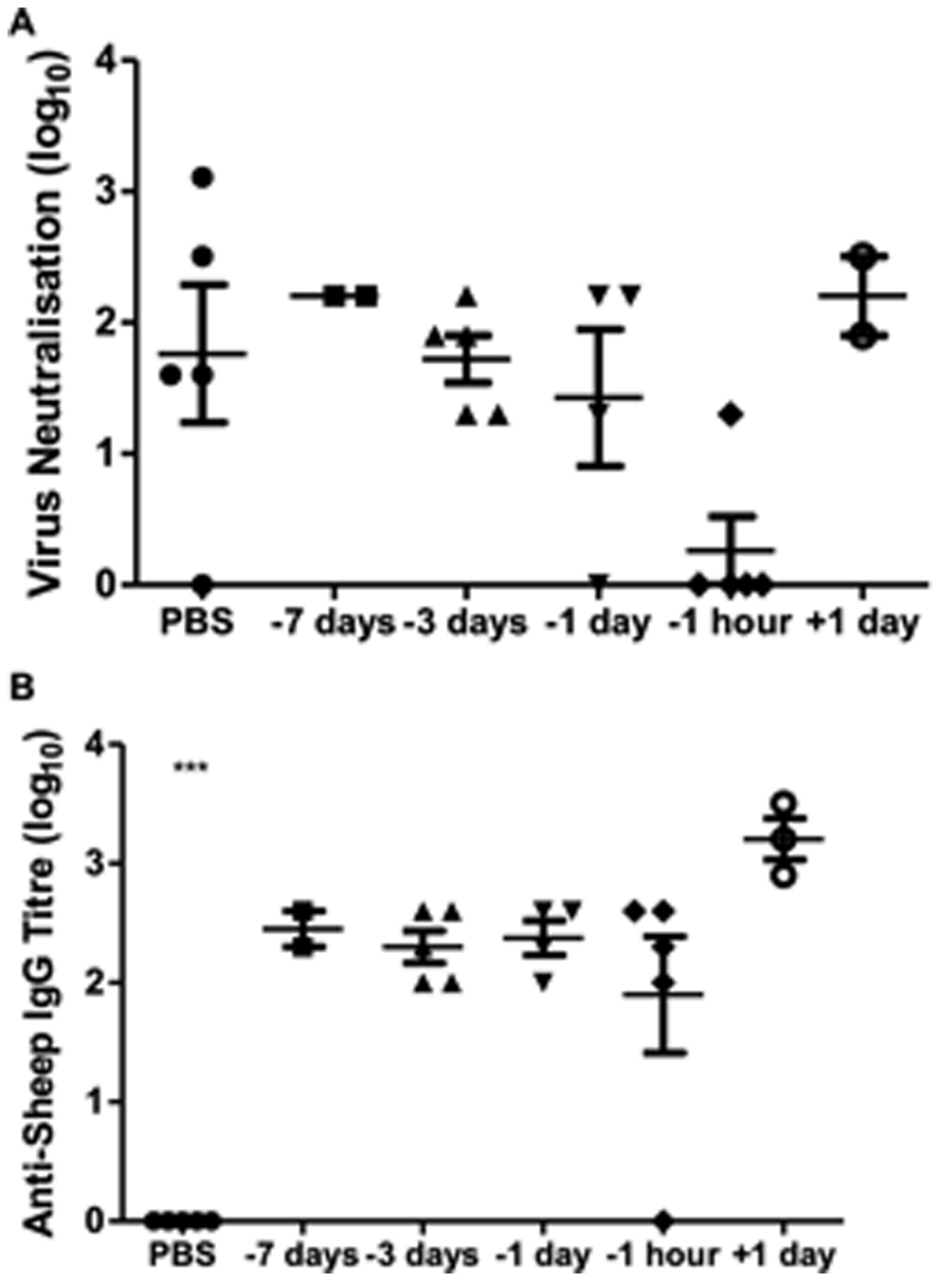
Antibody responses to ovine IgG and virus in ovine IgG protected mice. Lightly anaesthetized female BALB/c mice (n = 5) were given 125µg IgG from split virus-immunized sheep or PBS by the intranasal route one hour, 1 day, 3 days, 7 days before or 1 day after i.n. challenge with 10^2^TCID_50_ PR8. Endpoint antibody titres in the serum collected at day 14 p.i. were measured by (**a**) MN assay for reactivity to homologous PR8 virus and (**b**) ELISA for reactivity to sheep IgG. Results are representative of two separate experiments with individual titres and the mean ± SEM shown. ***P≤0.001 represent comparisons between antibody-treated and PBS control groups.

As antibody responses to influenza virus are commonly used as a correlate of protection in most species, we examined the effects of prophylaxis on the development of endogenous antibody responses to virus in mice using serum collected 14 days p.i. A strong antiviral antibody response was observed in the virus-infected PBS control mice (Figure 5a). However, circulating antibodies had markedly reduced MN titres in mice given neutralizing antibody one hour before virus challenge, suggesting immediate virus neutralization. No significant changes in antibody levels were seen for mice treated with ovine IgG 1 day, 3 days or 7 days before and 1 day after virus exposure, indicating that these mice were virus-infected. Since xenogeneic protein can also stimulate B cell antibody production [46], we evaluated the humoral immune response to sheep Ig in mice (Figure 5b). Despite no signs of adverse reactions, an anti-ovine antibody response was generated in all mice given ovine polyclonal IgG by the i.n. route. As expected, there was no evidence of antibody reactivity to sheep Ig in the PBS-treated and virus-infected control mice that were not pretreated with ovine antibodies.

## Discussion

In this study our objective was to evaluate the efficacy of passive immunization with antibody on influenza prophylaxis using a ruminant source of polyclonal IgG antibodies. Virus infection stimulates innate immune responses that promote clearance of influenza virus infections, but these responses can also contribute to inflammation and pulmonary pathology [25], [26]. Although passive immunization with ruminant antibody has been shown to be effective against influenza [47], little is known of its influence on the host innate immune response. Since hyper-induction of innate cytokines leads to acute respiratory distress, knowledge of the effects of passive antibody for influenza prophylaxis on cytokine induction is essential [48]. Here we generated polyclonal antibodies against influenza A/PR8/34 (H1N1) virus in sheep, which were then administered by the intranasal route to mice followed by intranasal challenge with homologous virus. We observed significant reduction of body weight loss, clinical signs and lung viral loads and identified that the ruminant polyclonal IgG antibodies were efficacious without induction of the host innate immune response. Thus passive immunization with ruminant polyclonal antibodies represents an additional therapeutic to the arsenal against influenza.

Whole inactivated virus was previously identified as an inexpensive and potent immunogen in animals and is used routinely to vaccinate many species, including poultry against avian influenza viruses [49], [50]. Split virus vaccines are currently used for immunization to target the viral surface glycoproteins HA and NA [51], [52]. We tested the immunogenicity of these influenza viral antigen preparations in sheep to generate circulating antibodies. Although both viral antigen preparations induced ovine antibody responses, the split virus antigen was more immunogenic in sheep, largely eliciting more neutralizing antibodies in the serum and whey as determined by the MN, HI and NI assays, possibly due to increased viral H and N proteins.

In our study, ovine influenza-specific IgG was superior to PBS at reducing clinical, scores, weight loss and lung viral loads in mice when given prophylactically up to 3 days before virus exposure. We also observed normal lung architecture in mice protected with ovine antibody pre-treatment with ablation of the neutrophil infiltrate in the airways at day 3 [data not shown], an immune response shown to predict gene signatures in lethal influenza with immune-related pathology [53], [54]. Intranasal delivery of bovine IgG was previously identified as being prophylactic for influenza in mice, when both specificity of antibodies and challenge virus were strain matched and administered by the i.n. route [22]. However, these colostrum-derived antibodies are only produced for a short period of time immediately after calving. The continuous supply of antibodies produced in the milk of dairy animals provides a potential bulk source of antibody. Furthermore, antibodies delivered directly to the upper respiratory tract allows both virus neutralization and clearance of virus infection. Antibodies instilled in the nares of lightly anaesthetized mice must be able to protect the upper respiratory tract from invading virus, and possibly gain entrance to the lungs to guard against lower respiratory tract infection. The mucosal route of delivery also minimizes the risk of host allergic-type reactivity to foreign proteins as it is systemically non-invasive. We have found previously, that repeated intranasal administration of ovine IgG to mice daily for 6 days does not induce adverse side effects, despite a neutrophil response in the lungs [data not shown]. However, it will be important to further assess the immunogenicity and safety of ruminant antibodies. Also the efficacy of i.n. delivery of antibodies in protecting animals against exposure to an aerosolized virus warrants future studies in order to reflect natural influenza A virus transmission.

Although the protective effect of ovine IgG was complete when a single dose of 125µg IgG was given one hour before virus challenge, this likely indicates immediate virus neutralization in the nasal passages as a strong endogenous antiviral antibody response was prevented. In contrast, control mice receiving PBS treatment 1 hour before virus challenge showed severe clinical illness, lost significant weight, and developed an antiviral antibody response to virus infection. To test the robustness of passive immunity we also examined the influence of timing on protective efficacy of ovine virus-specific IgG for influenza. Protective effects were demonstrated with 125µg IgG delivered to mice at 1 day and 3 days prior to virus infection. As such mice also produced an influenza-specific IgG antibody response, evident by ELISA using day 14 immune sera, this result suggests that the protective ovine antibodies were capable of clearing an acute virus infection in the respiratory tract. However, therapeutic use of IgG even 1 day after virus infection did not reduce viral load in the lungs or lessen clinical disease symptoms. This was an unexpected finding as others have reported therapeutic efficacy of antibodies, although protection was reduced with times beyond 24 hours after virus infection [55]. Higher doses of antibody with increased neutralization capacity may overcome this problem as has been observed in other reports of antibody therapy [56]. Further studies to elucidate the protective efficacy of ovine antibodies against lethal influenza virus doses would provide data on mortality rates.

For the first time passive immunization with ovine antibody was examined in mice for modulation of the innate cytokine response in the lung to respiratory virus infection. Type I IFNs and pro-inflammatory cytokines IL-1β, TNF-α, IL-6, and CXCL1 were shown to be rapidly induced by virus in PBS-treated control animals, whereas in antibody-protected animals their levels were mostly reduced. These results indicate that the protective effects mediated by passively acquired antibody before virus exposure influence the host’s innate cytokine responses.

Acute respiratory infections are responsible for nearly 4 million deaths every year, mostly of children and infants in developing countries [57]. There is a real threat that a pandemic caused by influenza could occur at anytime with dire consequences. Current control methods of vaccination for prevention of influenza virus infection and antiviral drugs for treatment of influenza are not ideal in a pandemic situation due to high manufacturing costs, delays in production and emerging resistance to antivirals. Therefore, adjunct options for control of influenza need to be developed. Use of convalescent sera from people recovered from H1N1 influenza in the 1918 pandemic was successful during a period when antivirals and antibiotics were not available [12]. Also, convalescent plasma was used to effectively treat H5N1 infected individuals in China [58]. Since the supply of human immune sera is scarce, particularly during a pandemic, an alternative supply is required. We propose the generation of anti-influenza virus polyclonal antibodies in ruminants. Such a strategy has several benefits over challenges of vaccination with strain-matching requiring updated formulations of virus antigens and the protracted time of several weeks to develop full immunity post-immunization. This approach represents a barricade control measure for influenza that could be prepared in advance and stockpiled for use during the intervening period between identification of a new virus strain and availability of a vaccine for influenza epidemics and pandemics. Here we provide proof-of-concept for use of ruminant antibodies. Our mouse studies clearly establish that ovine anti-PR8 antibodies can prevent and/or clear influenza infection. Polyclonal antibody preparations are associated with lower cost, rapid production times and suitability for low resource settings, making them ideal for use in regions likely to experience virus outbreaks during the crisis period of an influenza pandemic. Further development of ruminant antibodies with broad neutralizing capacity and evaluation in challenge studies with heterologous influenza virus strains are required to reveal their full potential.
